# World’s Columbian Dental Congress—1893

**Published:** 1893-10

**Authors:** J. M. Walker


### THE DENTAL REGISTER.

VOL. XLVII.] OCTOBER, 1893. [No. 10.

### SOCIETY PROCEEDINGS.

### World's Columbian Dental Congress,—1893.

SCIENTIFIC WORK BY SECTIONS.

Reported for the DENTAL REGISTER by Mrs. J. M. Walker.

In accordance with the general programme outlined in the prospectus issued by the General Executive Committee, the World's Columbian Dental Congress convened in first general session in the Hall of Washington, Memorial Art Palace, Chicago, at 11 A. M. August 14, 1893.

The Session was called to order by Dr. W. W. Walker, Chairman of the Executive Committee, who called upon Dr. J. Taft, of Cincinnati, to invoke the Divine blessing upon the deliberations of the congress.

Dr. Walker then introduced Hon. C. C. Bonney, President of the World's Congress Auxiliary, who opened the congress in an address in which he specialized dentistry as a conspicuous representative of an important movement of the present age, viz: the specialization of scientific pursuits. He said, in brief, that the old fields of research and application were so narrow that they were readily mastered, but by the marvelous development of modern civilization all this has been changed. Only a powerful glass can now trace the horizon of scientific attainment. In the swiftly developing evolutions of arts and sciences the great work of orderly differentiation has gone forward subdividing professional and all other pursuits, so that a mere general knowledge does not suffice in any department of science or of art. The general physician and surgeon can no longer be trusted to deal with the exact mechanism of the eye or the ear, nor with the important and delicate relations of the teeth. A lifetime may be spent, and the highest abilities and attainments be exercised in an apparently limited and specialized field. The old-fashioned tooth-carpenter, well named in his day and time, has become like the fabled Dodo, an extinct species, and we have in his place the modern doctor of dental surgery—accomplished, learned and skilled ; familiar with anatomy, physiology, pathology, histology, etiology, bacteriology, chemistry and metallurgy, as well as the practical operations of the art.

In conclusion, President Bonney congratulated the congress on the admirable programme offered and the international character of the papers to be presented.

Dr. W. W. Walker, Chairman of the Executive Committee, was then presented and addressed the congress briefly on the subject of the work accomplished by the Executive Committee, working hand in hand and shoulder to shoulder to accomplish for the dental profession that which places it where it should be placed—in the front rank of the scientific professions of the world.

Dr. Walker next introduced Dr. L. D. Shepard, President of the World's Columbian Dental Congress, the other officers of the congress, the members of the Executive Committee and the Committee of the Woman's Branch.

The resolutions creating the congress were then read by Dr. A. W. Harlan, Secretary General of the Congress.

The announcement was made that the Hon. John Temple Graves, of Athens, Ga., who was to have delivered the address of welcome to the foreign representatives was unable to be present owing to sudden illness.

Dr. J. Y. Crawford, who was called upon on very short notice, responded in a greeting noted for cordiality, warmth and hospitality. He said that he stood as the mouth-piece, not only of the dental profession of America but of the whole people, expressing the voice of seventy million souls, saying with and for them, "Be with us, among us, and assist us in furthering the grand work of the dental profession." He said that of all questions that address themselves to the civilization of the nineteenth century dental surgery stands at the head of the list, because it is not yet a universally recognized fact that the human family needs the good offices and attention of the dentist as they need their doctor, preacher, teacher or lawyer. With the further advancement of the profession civilization will be touched and benefited by the increase of the average of human life, becoming a potent factor in the perpetuation of the most remarkable civilization in the world's history. In extending a special welcome to the representatives from foreign countries, he said, that the ensign of the republic was not only an insignia of political and religious liberty, but a sign of the gracious hospitality extended to-day. He said the united sentiment of this great nation passes from me to you while uttering these words of welcome.

Letters regretting inability to attend, because of illness, were read from Drs. W. W. H. Thackston, Farmville, Va., and W. H. Morgan, Nashville, Tenn., two of the vice-presidents.

Dr. L. D. Shepard then delivered his inaugural address, in which he reviewed the history of the congress from its first inception. Dr. Shepard included in his address an outline of the evolution of the profession with the salient points marking its progress. He dismissed so-called ancient dentistry with very few words, pronouncing dentistry even as an art modern, and as a science essentially and altogether modern. He sketched vividly the conditions existing immediately preceding the establishment of the First Dental College, 1839; the publishing of the First Dental Journal, 1840; the organization of the First Dental Society, and the enactment of the First State Law regulating the Practice of Dentistry. The next great event in dental history was the most notable and beneficent discovery of all ages—that of anæsthesia, to which no other discovery or invention is comparable. In the next decade Colleges, Magazines, and Associations increased and multiplied rapidly. Of the next decade the most momentous discovery was that of the value of the cohesive property of gold, creating a new era in operative dentistry, the era of restoration—the parting line between antique mutilation and disfiguration and the subsequent devotion to beauty and typal form. This was the first great advance in practice, and was at once supplemented by improved instruments, the mallet, the rubber dam, and the engine. These combined wrought a complete revolution in practical dentistry. Dr. Shepard also described the ebbs and floods in the tide of prosthetic dentistry, the elevation of the standards of the Colleges, the adoption of Codes of Ethics, and the most recent advance in the retention of teeth or roots by the invention of the modern artificial crown and its corollary the bridge—the most distinctive improvement of the past twenty years. Later years are marked by an increasing interest in the study of the deeper causes of physiological function, and of pathological departure from the normal. Histological investigations are pursued with great thoroughness, and the dental tissues are studied by the microscopist with great courage and self-denial.

The theory of the bacterial origin of disease and antisepsis has engrossed much study and research, bringing more thorough acquaintance with ptomaines, and leucomaines, giving practical results in the sterilization of everything connected with operative work. Prophylaxis is developing along physiological lines in the observance of the laws of hygiene; the teeth, as parts of the general economy, sharing in the great benefits thus derived. In concluding Dr. Shepard referred to the great army of those "gone before," the present Congress being but the culmination of their patient, devoted, self-sacrificing work for God and man.

Dr. Shepard closed his address with the hope that this week may be so full of inspirations and valuable results that it shall be a mile stone in the march of dental professional progress.

---

To facilitate the scientific work of the Dental Congress eight sections met simultaneously, in as many different halls of the Art Palace, as follows:

SECTION 1. ANATOMY AND HISTOLOGY, R. R. Andrews, Cambridge, Mass., Chairman; Geo. S. Allan, New York, Vice-Chairman; F. T. Breene, Iowa City, Secretary.

SEC. 2. ETIOLOGY, PATHOLOGY AND BACTERIOLOGY, G. V. Black, Jacksonville, Ill., Chairman; E. S. Chisholm, Tuscaloosa, Ala., Secretary.

SEC. 3. CHEMISTRY AND METALLURGY, D. R. Stubblefield, Nashville, Tenn., Chairman; J. S. Cassidy, Covington, Ky., Vice-Chairman; E. V. McLeod, New Bedford, Mass., Secretary.

SEC. 4. THERAPEUTICS AND MATERIA MEDICA, F. J. S. Gorgas, Baltimore, Md., Chairman; N. S. Hoff, Ann Arbor, Mich., Vice-Chairman; J. E. Cravens, Indianapolis, Ind., Secretary.

SEC. 5. DENTAL AND ORAL SURGERY, T. W. Brophy, Chicago, Ill., Chairman; L. P. Dotterer, Charleston, S. C., Secretary.

SEC. 6. OPERATIVE DENTISTRY, Wm. Jarvie, Brooklyn, N. Y., Chairman; H. W. Morgan, Nashville, Tenn., Secretary.

SEC. 7. PROSTHESIS AND ORTHODONTIA, C. L. Goddard, San Francisco, Cal., Chairman; E. H. Angle, Minneapolis, Minn., Secretary.

SEC. 8. EDUCATION, LEGISLATION, LITERATURE, J. J. R. Patrick, Belleville, Ill., Chairman; W. H. Whitslar, Cleveland, Ohio, Secretary.

### MONDAY, AUGUST 14, 1893.

Section 1, Anatomy and Histology, organization deferred until Tuesday owing to delay in the arrival of the baggage of the Chairman, Dr. R. R. Andrews, containing his address.

Sec. 2, Etiology, Pathology and Bacteriology. This section was organized and Dr. G. V. Black, the Chairman, delivered an address of welcome, in which he urged the importance of the study of bacteriology, and the use of the microscope in making clear many of the mysteries of etiology and pathology. The section then adjourned until 2:30 p. m. Tuesday.

Sec. 3, Chemistry and Metallurgy, was not organized on Monday.

Sec. 4, Therapeutics and Materia Medica. This section was called to order and limited to a brief address from the Chairman,

Dr. F. J. S. Gorgas, and then adjourned to 2:30 p. m. Tuesday.

Sec. 5, Dental and Oral Surgery, and Sec. 6, Operative Dentistry, not organized on Monday.

Sec. 7, Prosthesis and Orthodontia. Dr. C. L. Goddard, of San Francisco, Chairman of the Section, read an opening address giving a brief history of the development of prosthetic or mechanical dentistry, noting the decline of mechanical dentistry upon the introduction of vulcanite work, and its revival upon the introduction of crown and bridge work, the culmination of mechanical dentistry.

He also contrasted the use of the old rubber bands and wooden wedges with the modern system of orthodontia, a comparatively new branch of prosthesis, or rather of surgery—artistic surgery—reducing deformity to beauty, and requiring all the skill and delicate perception of the artist both in color and in form.

After a cordial greeting, extended to all, the chairman pronounced the section adjourned to 2:30 p. m. Tuesday.

Sec. 8, Education, Legislation and Literature. This section was called to order by the Chairman, Dr. J. J. R. Patrick, who delivered a brief address of welcome to the members present, referring briefly to the importance of the questions to be brought before them for discussion. Adjourned to 2:30 p. m. Tuesday.

TUESDAY, AUGUST 15. SECOND DAY, GENERAL SESSION.

The meeting was called to order by the Chairman, Dr. L. D. Shepard, who called for representatives from foreign countries who were not present yesterday. Dr. I. L. Secher, of Copenhagen, representing Denmark; Dr. K. Takayama, on behalf of Japan, and Dr. R. H. Robertson, Honorary Secretary from Canada, responded and were cordially welcomed.

The chairman then introduced Dr. Otto Zsigmondy, of Vienna, Austria, who read an illustrated paper entitled "Congenital Defects of the Enamel," of which we give a brief outline: He defined "erosion," or "atrophy" as a frequent anomaly in enamel-development, in which the outer coating of the tooth is unequally distributed, and of only superficial depth, in pits either isolated or occurring in more or less regular rows.

When the pits are confluent, furrows are developed which may embrace the crown like a ring. At times several of such furrows are found arrayed in series, one above the other, [furrows or wavy enamel]. In other cases the enamel appears as if sown with small pits. In still other cases the enamel seems to be entirely absent at certain points, and the dentine itself comes to the surface. The defect may also affect the cutting edges of the front teeth. In the pits the enamel often has a rough, uneven surface, a more or less yellowish or brownish color, and lacks the proper polish and normal transparency, while the enamel in the immediate neighborhood of the spots has all the characteristics of the perfect tissue. In those teeth in which the dentine is exposed, or covered by a thin layer of enamel only, the enamel of the normal portion of the tooth is apparently thickened, but cross sections of such teeth show that there is no increase in thickness above the normal. Spots entirely devoid of enamel are very rarely found. The dentine of these defective teeth is also very faulty in structure and conducive to the rapid progress of caries. It is characteristic of the defects in question that they are always symmetrical, so that corresponding teeth in the upper and lower jaws on both sides are similarly affected. The defects occur in teeth the development of which corresponds to the same period of time, and in the teeth they are limited to such portions as correspond in degree of development. The situation of the defects and their distance from the lateral surface of the crown accordingly vary in the different orders of the teeth. At times, if the interference with the development occurred at a very early period and no interruption in normal development took place later, it is only the first four molars, the first of the permanent teeth to harden, which show signs of abnormality. The defects are then found at the apices of the cusps only, which, in consequence, seem as if worn down. If the interruption of normal development occurs at a later period, when the formation of the enamel of the cusps of the first molars is further advanced, these teeth show the defect more in the direction of the root. In this case, besides the first molars, the central incisors likewise show defects in the enamel of the teeth which come next in point of time of calcification.

If the disturbance be repeated once or oftener, a corresponding series of furrows, or pits, will be found in the enamel of the teeth which were in process of calcification at the time of interruption of development. If a furrow be found in the first molar near the edge an analogous defect will appear half way up on the crown of the central incisor. The cuspid will show the defect nearer the point of the cusp, while the first molar and the teeth which undergo calcification later will be free from defect.

Calcification of the upper jaw proceeds as follows:

1. The central incisor.
2. Cuspid.
3. Lateral Incisor.

This noteworthy circumstance, as yet inadequately studied, determines why we find the lateral incisor developed almost normally, while defects are apparent in the enamel of other teeth of the upper jaw.

The first molar is but rarely the seat of typical defects, the second molar still more rarely, and no instance of such defect has ever been observed in the two last molars. Certain writers maintain that the milk teeth never exhibit typical defects. This, however, is a mistake. Temporary teeth are occasionally observed which resemble permanent cuspids and molars in defects of the enamel. As, in the majority of cases the entire denture is involved, the cause of this anomaly must be sought for in general diseases of the organism, the effect of which as regards the other tissues of the body has vanished, while its influence has become permanent in the teeth.

Rachitis, scrofulosis, syphilis, the exanthemata, convulsions, meningitis, grave attacks of suffocation, as, for example, from whooping-cough in early life, have been the alleged causes of the disturbances to the normal development of the teeth in their follicles. In view of the literature dealing with the subject, it is rather astonishing that the microscopic examinations of teeth so affected should have been almost entirely neglected, as this must form the basis for the solution of the question of the cause of the deformities.

Dr. Zsigmondy pronounced the nomenclature, used in describing these defects, very faulty. Terms which are appropriate for a great many cases, such as, "wavy enamel," "honey-combed teeth," "furrowed teeth," and similar expressions, are not suitable in every case. The descriptive appellations "syphilitic" or "rachitic" teeth are to be rejected, because it is doubtful whether the diseases are the real cause of the defects, and if they are a cause they can only be so in a small proportion of cases. The term "atrophy" is likewise incorrect. The expression "erosion" is no less to be rejected. Erosion signifies loss of substance by mechanical force. The term erosion is properly applied to the notch-like grooves found on the necks of teeth, and which have been produced by the use of gritty tooth-powders, etc.

He said, I shall take the liberty of offering the following:

Where individual organs or part of organs are defectively developed because of external or internal noxæ, pathological anatomists are wont to employ the term *hyperplæsia* to express that condition. We may accordingly speak of a hyperplasia of the enamel.

May I be permitted to express the hope that this term may soon find a place on the literature of the subject.

The paper was illustrated by charts and specimens of defective deciduous teeth, etc., and elicited frequent applause.

It was discussed at some length by Drs. J. J. R. Patrick, G. V. Black, L. L. Davis, George Cunningham, (England) and others.

Dr. Patrick said, we all know if there are any congenital defects in the enamel of the deciduous or permanent teeth, they must have their origin when that enamel is formed; and if hereditary disease is the cause of such defective enamel, it must necessarily affect the deciduous teeth first, that is the point I wish to make. All eruptive diseases during gestation must necessarily affect the enamel of deciduous teeth if the disease takes place during the formative process of the enamel. Can it be otherwise? I have already put on record three very remarkable cases bearing upon that subject, the effect of eruptive diseases on the enamel.

Dr. Zsigmondy said, I have observed just the same defects in the deciduous teeth as in the permanent set. I have past around a bottle containing two cuspids of the deciduous set, where defects appear just as in the permanent teeth, and which exhibit the characteristic microscopic structure. I have not seen it in the deciduous central incisors; these teeth are very much more liable to decay; but you will find here, if you examine the cuspids which I have exhibited, just the same effect as you find in the permanent teeth. I have made microscopic slides, and I find the same line of interruption as in the permanent set. This was not, however, in a syphilitic case.

Dr. Patrick—What was the age of the patient when those teeth were extracted?

Dr. Zsigmondy—Ten years.

Dr. Cunningham called attention to the fact that in the paper the question of the cause of these defects is not entered into—it is a statement of observed facts, and the subject is far from exhausted. He suggested that the paper be referred to the appropriate section for further discussion after inspection of the specimens and careful reading of the paper.

He said, with regard to this line of interruption which he mentions: this is a new fact brought before us in a very interesting way. So far as the microscopic appearance is concerned, no other defects appear upon the coronal portion of the tooth; yet when you examine the sections through the microscope you find this line of interruption appearing. It must have been due to the same cause, because it is a much larger defect.

Those who are familiar with the development of the cap of dentine will see further that the calcification is not an even and straight line, but it is wavy, and the waviness will account for that defect.

In studying this question, you will find the defects may be little or great, and the duration may be short or long; we find a great variety of defects.

In regard to the nomenclature of the subject Dr. Cunningham said, there is no doubt but that the term which has been suggested, "hyperplasia," is in conformance with recent researches in pathology.

Further, we have had suggested, in addition, that in talking of enamel defects, to distinguish them from hyperplasia, which might be a term also applied to the root or cementum of the teeth, the use of the term coronal hyperplasia, not confining ourselves to the enamel, because it is perfectly evident that these interruptions of development affect the dentine as well as the enamel. In giving it a name we must take the name, if we possibly can, that will appeal to the whole structure.

Dr. C. C. Carroll objected to the use of the term "congenital" in the title of the paper, as it had led him to expect what was conspicuously omitted, viz: a discussion of the cause of the disease, a definition of the hereditary line, and an indication of the character of the disease.

The paper was then referred to Section No. 2. The Secretary-General then read several notices and the Congress adjourned until Wednesday, August 16th, at 12 o'clock.

WORK OF THE SECTIONS, TUESDAY, AUGUST 15.

Sec. 1 was organized, and the chairman, Dr. R. R. Andrews, read an earnest address of welcome. Of the position rightfully to be occupied by dentistry, he said, dentistry should not be one of the fugitive and obscure callings, irresponsible and independent; it should seek to fill an honorable place in the ranks of the great medical fraternity, where I believe it justly belongs. Our place is, and ought to be, among the specialties of the great guild of physicians. The occulist, the aurist, and the dentist should stand side by side, part of the great body of scientific workers who heal the sick.

Of the contributions of dentistry to science and literature, he said, our histological and pathological literature, our mechanical inventions, our ever-increasing discoveries with chemistry and the microscope, all show with what far-seeing study and native originality our students apply themselves and claim their places among the scientific explorers and molders of scientific thought.

After words of cordial greeting he closed, saying, let us have a free and open arena for discussion; let fraternity and good will dominate our council, and we shall mark the work of our section, of this Congress of 1893, as one of the most productive of good results we have ever held.

The first paper read before this section was entitled, " What has Dentistry to Demonstrate Against the Hypothesis of Organic Evolution?" by W. G. A. Bonwill, D.D.S., of Philadelphia, Pennsylvania.

The paper was an elaborate treatise on the geometrical arrangement of the teeth, together with their sizes and shapes, showing all to be based upon the equi-lateral triangle.

The argument was based upon a statement in thirteen propositions of mathematical principles as applied to the construction of the human jaw, which he claimed was necessarily perfect from the very beginning, the only changes being in the line of retrogression, due to civilization and man's unwise interference.

The mathematical principles involved cannot be reduced within the limits of an abstract, while the working model and drawings with which the paper was illustrated are essential to its comprehension.

The paper was discussed by Drs. Geo. A. Mills, J. J. R. Patrick, W. X. Sudduth, Eben M. Flagg, (Paraguay), L. P. Haskell, C. N. Peirce, and Z. Schwarze, (Leipsic).

Dr. Haskell said, that he had, for sometime, been adopting the principles laid down by Dr. Bonwill, in the arrangement and articulation of artificial teeth, and he finds that they apply with admirable results.

He referred to certain facts which he had verified in a very large number of cases, viz: that the alveolar process is shorter on the left side than on the right, while the teeth are higher and more prominent on the left side, while the right side is further from the median line than the left side. Dr. Haskell adopts Dr. Talbot's explanation of these facts, viz: that in mastication the jaw moves from right to left, and that though we chew on both sides of the mouth, it is more common to bite off food on the right side of the mouth. The result of all this is the gradual change in the alveolus noted.

Dr. J. J. R. Patrick said that he did not feel capable of discussing the question, as he was not sufficiently versed in mathematics, and then proceeded to analyze Dr. Bonwill's series of propositions, controverting each one in turn with rather bitter sarcasm and ridicule. The first proposition he pronounced "rotten to the core." The jaws he classified as parabolical curves rather than equilateral triangles, the jaw in its movement describing the arc of a circle. The human jaw is a lever of the third order, the power being the masseter muscle placed between the fulcrum and the anterior portion of the jaw which is to be moved at the angle of the ramus. As to the principle that the equilateral triangle underlies the shape of every tooth, Dr. Patrick said, you cannot get an equilateral triangle out of any one of them. He said, it is a bold man who will say that the Creator has made any thing wrong, or that he could not produce a human being on the square just as well as he could on an equilateral triangle; I am only sorry that he has not produced more men on the square than he has.

Dr. Bonwill said in reply that although he had heard but very little of what Dr. Patrick had said, he could see plainly in his countenance animosity and ridicule. He said, all that I ask you to do is to follow me in every line that I have given here and prove whether I am right or wrong.

Dr. C. N. Peirce agreed with Dr. Patrick that Dr. Bonwill's conclusions were inconsistent with his text, and could not be legitimately drawn from it.

Dr. Sudduth agreed with Dr. Peirce, but considered that Dr. Bonwill has discovered the law that governs the articulation of the human teeth, as we find them at the present time, a law that, taken and applied to this articulation that he has developed, gives us the best and most practical articulation that we can make for artificial dentures, so far as my experience goes.

He said, there is a principle here that will be a benefit to every dentist who will study the law and apply it in his practice, as far as possible, as Dr. Haskell has testified he has done.

Dr. Schwarze, Leipsic, Germany, then made a few remarks, approving of Dr. Bonwill's method, and saying that it was almost the same as was being followed in the universities at Berlin and Leipsic.

The section then adjourned to meet Wednesday at 2:30 p. m.

SECTION 2. ETIOLOGY, PATHOLOGY AND BACTERIOLOGY.

Called to order by Dr. G. V. Black, chairman, at 2:45 p. m.

Discussion upon the paper of Dr. O. Zsigmondy, on "Congenital Defects of the Enamal" read before the general session at 12 m. and referred to the section for discussion was announced as the first order of business.

No one responding, the chairman stated that if any one should afterward desire to discuss the paper, it might be taken up at the completion of any subject brought forward for discussion.

Annie Felton Reynolds, D.D.S., of Boston, Mass., read a paper entitled: "Adenoid Growths and other Diseases Incident to Primary Dentition."

After reciting briefly and succinctly the well known facts relating to the primary dentition, the indications of premature and delayed eruption, the bearing of different conditions and diseases upon the process and their effect upon the deciduous teeth, the paper took the ground that not difficult dentition, as held by the older practitioners and the laity, but a rachitical tendency, is the predisposing cause of infantile convulsions, although an inflamed and tender gum may precipitate the paroxysm.

The time of dentition is a period of great functional activity, for besides the eruption of the teeth, the follicular apparatus of the intestines is undergoing active development in order to prepare the system for a radical change of diet. As a consequence, gastro-intestinal disorders and nervous symptoms are frequent. The presence of undigested and indigestible food in the stomach and intestines is a very fertile cause for convulsions. Overfeeding, with proper food even becomes an exciting cause, especially during hot weather. Cutaneous eruptions are very common at this period of the child's life, and may appear contemporaneously with the cutting of a tooth. Salivation is of frequent occurrence in children, apart from the usual flow which accompanies dentition, and is a characteristic symptom of ulcerative stomatitis.

Abnormal growths in the naso-pharyngeal cavity are very likely to appear at this period, causing not only morbid conditions of the whole respiratory tract and middle ear, but also pathological changes in other and remote organs of the body. These abnormal or adenoid growths were minutely described as to color, form, size, mode of attachment, etc., the symptoms being grouped under five heads: respiration, secretion, speech, hearing, and general condition. The respiratory effects are direct and indirect, the former being seen in the obstruction of nasal and the establishment of oral respirations. The general state of the patient is affected in many ways. The imperfect and unnatural breathing creates anemia, which may lead to lack of development of the bones of the face, deformities of the upper jaw, irregularities of the teeth, mis-shapen chest walls, deafness, and defective speech. In mouth-breathing of long standing, the superior dental arch ordinarily is contracted and the roof of the mouth elevated. In a child of two years, the average age when the trouble begins, although the growth may be found in an infant of a few days, little else than difficulty of breathing, especially at night, is noticed. An ordinary cold may have been the initial cause. The child soon shows a tendency to repeated attacks of cold in the head, which increase in frequency and duration, accompanied by considerable nasal discharge, which may be entirely absent when the child is free from acute attacks. Deafness, in the majority of cases, is soon added to the list of discomforts, and by the time the child has reached the age of six, eight, or ten years, the characteristics of a typical example of adenoid vegetation are as follows: the child is a mouth-breather, its facial expression is dull and stupid, oftentimes idiotic. The open or half-closed mouth is one of the most constant symptoms. Nasal breathing is interfered with during the day, but at night the child never sleeps soundly; it assumes unnatural positions in bed, the slightest noise will awaken it, and during the day it is languid and irritable. The majority of such children are deaf, and their voices are stuffy and thick. Again, nervous symptoms are sometimes associated with this disease, and abnormal sensations in the head, and pressure in the upper and back part of the cranial cavity.

Once established, some of these results remain through life; others may be removed or corrected by clearing the air passage; all of which could have been prevented by an early removal of the growths, whereby natural and free breathing would be established through the nostrils. There is a definite relation between these conditions and the V-shaped palatine arch, which has not been appreciated in considering the acquired causes of the irregularities of the teeth in children, and which should be emphasized. The high arched palate always presents in typical cases of this complaint; moreover, it is one of the earliest mechanical results of obstructed nasal breathing. Mouth-breathing, being unavoidable in these cases, a constant pressure of the air inside the buccal cavity, gradually but surely forces the hard palate upward. From the simple vaulting of the palate in the very young it may go on to marked deformity of the jaw and great irregularity of the teeth; the central incisors suffer most, being in some instances rotated so that they stand at almost a right angle to the jaw. Many a child has had healthy teeth extracted, and has worn for months an ingenious mechanical device for correcting a crowded and irregular denture, while atmospheric pressure was constantly working against the efforts of the dentist. Whereas, if the growths were first removed, the labors of the dentist would have been lessened, since the establishing of free nasal breathing would remove the atmospheric pressure, and the teeth come more easily into place. The time to operate for the removal of these growths is from the age of a few weeks to that of sixteen years, the earlier the better for the patient; the operation is not difficult, and, properly performed, is not attended by danger to the child. The growths do not recur, and after their removal the breathing at once becomes quiet and regular, the mouth remains closed, the lungs expand with the increased supply of air, and the whole physique immediately responds in a marked degree. The child, hitherto called dull and stupid, now makes rapid progress, and his comrades soon cease making a laughing-stock of him. As dentists we have ample opportunities to diagnose this disease, which, so simple in itself, may be said to be malignant in its results, and we should realize our responsibilities in the matter, since by our neglect to cause the removal of these growths there is a risk of permanent damage to important functions, which may embitter the subsequent lives of these little ones, "no one of whom should perish."

Dr. Thomas Fillebrown, Boston, was called upon to open the discussion of this paper. He said, that much good was resulting from the treatment of these abnormal growths, and he was satisfied that a large proportion of the symptoms described, and so frequently seen in children with mouths so habitually open, are the result of growths in the pharanyx. He said, that he could not agree with the essayist as to the causes of the high vault and narrow arch. He thought that instead of being an elevation of the median line it was rather a depression of the sides, the unsupported weight of the teeth, when the mouth is habitually open, depressing the sides, narrowing the arch, and crowding the central incisors out of position.

Dr. E. S. Talbot said that he also differed with the essayist in regard to the cause of the "high arch." The arrest of development of the tissues is the result of a hereditary neurotic condition.

The presence of adenoid growths in the nose does not cause mouth-breathing, the high vault, and the contracted arch, but they are all one and the same thing. That is to say, a person who has had adenoid growths has a high vault, as we understand it, and a person who has a high vault has adenoid growths, contracted jaws, arrest of development of the face and upper jaw, a deformed dental arch.

Contraction is the arrest of development. The arrest of development stops at the sixth or seventh year, and that is the reason why we have adenoid growths and hypertrophy of the mucous membrane at that time; and the deformity of the dental arch is the result of this arrest of development. We are told that at that time the brain has gotten its growth.

He said, I do not know, nor does any one else, why the brain ceases to develop at that time, why in the case of an idiot the brain ceases to develop at that time, and why in the case of a genius, a Paderewski, a Blind Tom, a Michael Angelo, and most of our great men, why their brains cease to develop. But we do know, and it must now be settled as a fact, that these conditions are noticed among neurotics and degenerates. The specialist in diseases of the nose will tell you that about fifty per cent. of his patients have these conditions: deflected septa and excessive development of the turbinated bones. I am very positive of the points I have given you in a general way.

The next paper by P. Macarovici, M.D., Jassy, Roumania, was then read by the secretary of the section, Dr. E. S. Chisholm, it was entitled: "Pulpitis Chronica Idiopathica."

Pulpitis chronica idiopathica is a new formation of dentine, a dentine neoplasm. This pathological process arises in three ways:

1. By the proliferation of one or several cells of dentine tissue, forming a nodular growth which exerts pressure upon the nerves of the pulp. These nodules are situated on the inner wall of the pulp-chamber.

2. By a sort of obliteration of the pulp-chamber, whence the pulp beginning at the periphery is transmitted into secondary dentine, a pathological process most frequently found in teeth which are worn down or have become carious, usually in teeth affected with a low degree of inflammation.

3. By a new formation, having its origin in the pulp itself, and not connected with the walls of the chambers but free, either in the center or at the periphery of the pulp. This occurs rarely in teeth whose cusps are entirely unaffected.

Bertin was the first to observe that these new formations are of two kinds, an *adherent* and a *free* species, and he was hence the forerunner of Salter in his discourse, in that he made it easier for the latter to prove that these new formations of dentine are the result of a *pathological process*, although Bertin had described them only from the standpoint of anatomy. The older surgeons regarded this diseased condition of the teeth as normal.

According to Hyrtl, ossification of the pulp, or deposit of phosphoric or uric-acid salts, is the cause of the obliteration.

J. Bruck, Jr., says that he has found dentine formation in healthy milk-teeth with unabsorbed roots, and also in healthy permanent teeth, and adds that the size of the concretion was equal to a mustard seed. (Bruck, Jr., "Contributions to the Histology of the Teeth," 1871, p. 2.)

I have myself observed a single instance of such formation in a permanent, apparently healthy tooth, not worn at the crown nor not carious. The size of the new formation was equal to hemp-seed.

The theory of Dr. Macarovici as to the process of formation of these concretions is as follows:

The dentine fibers or fibers of Tomes are normally, so long as they are secluded from contact with the atmosphere, in my opinion, nothing else than lymph, which is derived from the pulp, traverses the dentine canals, and also penetrates into the enamel; they serve for the nutrition of the dentine and enamel, and partially also for that of the cement, where they may be regarded as auxiliaries of the alveolar periosteum. This fluid has the property of becoming solid on contact with the air, being fluid only while in transit from the pulp to the enamel, through the canal of the dentine. At the instant of contact with the atmosphere the fibers of Tomes solidify at their superficial extremity, forming an elastic membrane provided with minute pores, which allows of the transudation of a small portion of the still fluid lymph, and which in cases of fracture of the tooth, serves for the formation of the callus. If the solidification proceeds until finally the pores are obliterated, such solidification involves the entire extent of the fibers from their peripheral termination to their origin in the pulp.

It is my firm opinion, and it is shared by several authors, that the inner wall of dentine looking toward the pulp and surrounding the same, continues to grow after development of the teeth is complete, and that hence hyperemia brought on by intense emotion, violent movements, and other causes, may from the mineral substances contained in the blood bring about a hypertrophy of the dentine, which causes a narrowing of the canals of the dentine at their mouths in the walls of the pulp-chamber, and thus interferes with the entrance and circulation of the lymph, (fibers of Tomes.) The mineral substances are thus retained in the pulp, where they undergo evolution into dentine, cement, and enamel, which are intermingled, or else unite in groups of one or several homologous cells. This process has been verified by the observations of several authors, who state that they have found enamel, dentine, and cement in the new formation.

Notwithstanding the fact that lymphatic vessels have not yet been shown to exist in the pulp, it is nevertheless certain that lymph must be present in the pulp.

This theory of the fluid nature of the fibers of Tomes is substantiated by the formation of callus after fracture of the teeth. From whence comes this callus if not from the secretion of plastic lymph? And if the fractured portions of the crown contained no fluid in themselves, how could the lymph, which is supposed to come from the pulp, unite the fragments, which would in this case be toward one another as foreign bodies?

This theory also explains the great sensibility which exists on touching with a pointed probe a point in the line of junction between the dentine and the enamel, whereas the sensibility is much less at any point of the dentine itself. The hyperemia, which may be the cause of an inflammation of the pulp, determines the place of dentine formation. If, for instance, the inflammation is intense, the entire pulp is implicated, and the concretions come to be free in the chambers, and are of different size and form. If the inflammation is less intense, it becomes limited to certain parts of the pulp, only the apex or the lateral walls. In these cases the mouths of the canaliculi only are narrowed, which, correspond to the site of the inflammation, and the newly formed dentine is deposited in that spot and forms a mass in connection with the fibers of Tomes as well as with the dentine-canals.

Dr. Grevers, Amsterdam, said that he regretted that the essayist had not accepted the term adopted by the International Medical Congress, (Berlin, 1890,) for these hard formations in the dental pulp, viz., "odonticles." They are not always dentinal in character, but often mere chalky formations; neither were they altogether idiopathic. The term "pulpitis chronica *concrementalis*" would be better than *idiopathica.*

There being no further remarks the chairman passed the paper, subject to being called up again if so desired.

The section adjourned at 4 p. m.

### SECTION 3. CHEMISTRY AND METALLURGY.

Dr. E. M. Rockwood, of Iowa City, Iowa, read a paper entitled: "The Study of Chemistry in Dentistry."

The writer alluded to the storm of indignation aroused by the attempt of the census-makers to include dentistry in the mechanical trades, but thought that in the lack of preparation of the average student there might be found some reason for this apparent injustice. A trade may be learned with little cultivation of the perceptive faculties or of the reasoning powers. The study of chemistry leads the student to observe carefully and to draw conclusions from his observations. A certain amount of didactic instruction is necessary. The terms used must be given, the general principles of the Science must be explained, and the methods of the work described.

Work in the laboratory should commence as soon as possible. There the learner becomes familiar with the action of re-agents and with the various manifestations of chemical force. His hand is trained in the manipulations necessary for a successful experiment. He becomes acquainted with the common terms of the science, such as acid, base, and salt. By handling and testing these he learns by experience the methods of preparation and properties of many of his materials, like nitrous oxide, chlorine, and peroxide of hydrogen. The metals and their compounds offer a wide field for study. By the use of the blow-pipe the changes are noted which are produced by the influence of heat, as well as the action of fluxes. The effect of the oxygen of the air upon the metals at different temperatures is also learned. The subject of fermentation is one of interest to the dentist; and laboratory experiments will illustrate some of the most common forms, alcoholic, lactic, and butyric, and show the substances produced by the process. In connection with these simpler forms of fermentation, the putrefactive changes can be shown, with the action of different antiseptics and their comparative powers of checking the growth of organisms, which are the cause of putrefaction. The chemical action of each of these antiseptics must be clearly held in mind in order to be able to judge their suitability in particular cases. The same might be said of deodorizers and disinfectants.

Many points in physiological chemistry can be practically demonstrated in the laboratory. A complete analysis of tooth-substance should, of course, be made, and the properties of its constituents examined, not only as obtained from the tooth but also in the pure state. A thorough course should be taken in salivary analysis. This is as essential for the dentist as one in urinary analysis for the practitioner of medicine. The normal constituents should be so isolated, and a sufficient number of pathological specimens should be tested to gain a working knowledge of the subject.

Many of the common medical agents are chemical compounds, some of them administered for their chemical action, but all of them capable of acting chemically upon other substances which may be put into the same prescriptions. How many prescriptions are rendered inactive or positively harmful by an improper choice of its ingredients. It is true we can go to our books and find the necessary formula, just as we could consult our libraries to learn the proper course of action if an artery has been cut, but these fundamentals should be learned, so that they may be immediately available when they are needed. Such a knowledge can only be attained through the familiarity with such substances which comes from personal observation. This might also be said of the poisonous medicines and their antidotes, the latter owing their efficacy, for the most part, to their chemical properties.

Improvements must come largely either from the dental profession or from the manufacturer; the former is often unable to furnish them, and if they come from the latter they are immediately covered with patents. One aim, then, to be attained by a dental education, as in others which fit men for practical life, is to give such training as will enable a man to improve existing methods and invent new ones.

When the term of study is lengthened, as it must be to keep pace with the advances of the science, we shall be able to attain nearer to our ideas of perfection than we now can. Questions are arising daily which need to be answered by research. New remedies are being continually recommended, or quite often old ones or combinations of old ones under new and awe-inspiring names. What is their composition? Is their action chemical? Will it probably be beneficial or injurious? The decay of the teeth should be better understood. What produces favorable or unfavorable conditions? What is the action of the many disinfectants in use? And when should the different ones be employed? Do the sulphids or oxids of the metals which may be formed on amalgam fillings influence the decay, and if so in what manner? No one who is conversant with the many substances employed for cements and fillings believes that perfection has been reached in this line, even if he is able to use them all skillfully. The perfect filling is yet to be discovered. There is also much to be learned of the alloys. Do the metals cause an electrical action when the amalgam has set in the tooth so as to hasten or prevent its decay? The study of the influence of small quantities of a metal upon alloys needs investigations. Do the minute amounts of gold and platinum often used, one-tenth to three-tenths of a per cent. for instance, have a decided effect upon their properties? Tests should be made to determine the best combination, and the proper proportions of these in dental alloys. There is a field for observation, too, in the action of the mercury contained in amalgam fillings and in the coloring matter of plates, as to its state, and whether it produces the effects often ascribed to it upon the tissues of the mouth. Every dental practitioner can furnish queries from his own experience, and it is needless to give others; their number is practically limitless.

As we see the work in chemistry which is being done in some of our dental colleges, I think that we can conclude that its importance is becoming better appreciated, but there is still room for improvement. There is no reason to doubt that in the future it will occupy a prominent place among the studies which will best fit the dental student for his professional work.

This paper was passed without discussion, and the section adjourned to 2:30 p. m. Wednesday, August 16.

### SECTION 4. THERAPEUTICS AND MATERIA MEDICA.

This section was called to order at 2:30 p. m. Dr. F. J. S. Gorgas, of Baltimore, in the chair.

The first paper on the list for this section entitled, "A Method of Inducing Local Anæsthesia by Cocaine," by Dr. Caracatsanis, of Athens, Greece, was read by the chairman in the absence of the author, and later in the session was again read by the author himself.

He spoke of the untoward results often following the injection of cocaine and proceeded to explain his method of inducing local anesthesia as follows:

I begin painting the gum next the tooth to be extracted with an instrument wrapped in cotton, dipped in a solution of phenic acid, 2 to 1,000, which I have heated. This is followed by the application of the salt of cocaine by means of a pledget of cotton impregnated therewith. As soon as the gum shows signs of insensibility I commence to separate it slowly from the tooth by means of a bistoury. I insert into the space, thus effected, pledgets of cotton impregnated with cocaine as before. As the anæsthesia advances I enlarge the opening to a depth of about one centimeter on the buccal as well as on the lingual surface. I direct the patient to abstain from swallowing the saliva, to avoid all absorption of cocaine. I take good care not to forget the cotton pledgets placed between the gum and the tooth. After assuring myself by strongly making pressure on the parts with a steel instrument I have my assistant spray the parts with a mixture composed as follows:

| | |
|---|---|
| Chloroform | 25 grams. |
| Sulphuric ether | 40 grams. |
| Menthol | 3 grams. |
| Cocaine | 1 gram. |
| Essence of Mint | 1 gram. |

I extract while the parts are being sprayed. The resulting anæsthesia is absolutely complete; the only condition in which I have failed to produce it being the existence of inflammation, or periostitis.

To convince you thoroughly I am prepared to make an experiment with my method before your honorable congress.

Dr. Roberts spoke of the favorable results he had obtained in the use of tropacocaine, producing longer and more thorough anæsthesia, practically without any unfavorable symptoms from a 2 per cent. solution. The greatest objection to its general use is its cost, twenty cents per grain.

Dr. Seymour uses two drops of a twenty per cent. solution of hydrochlorate of cocaine and one drop of Merck's carbolic acid, as a local application, for five to ten minutes, by the use of which he is able to extract teeth without any perceptible pain.

Dr. Roberts considers from a two per cent. to a four per cent. solution of cocaine, antisepticised with chloral, practically safe and absolutely instantaneous, judged by his own experience in between two and three thousand extractions.

At this point Dr. Caracatsanis was introduced and read his paper, followed by the chairman, who, in the absence of Dr. Anthony Bleichsteiner, of Graz, Austria, read his paper on "Cocaine Injections for the Production of Anæsthesia."

Dr. Caracatsanis spoke of the paper on the same subject which he read before the International Dental Congress, Paris, 1889, in which he summed up the results of over three thousand injections of 5 per cent. solution of cocaine hydrochlorate. The present paper sums up his conclusions after more than fourteen thousand injections, using only the 3 per cent. solution since March, 1892, with corrosive sublimate for the sterilization of the solution.

He says: At first I renewed the solution immediately before every injection, but when I came to execute every extraction with cocaine anæsthesia, I prepared the cocaine solution in quantities of ten fluid-grams each, and I used for the sterilization of the solution, corrosive sublimate.

I took ten grams of sublimate solution, containing one gram of sublimate to five thousand grams of distilled water, and dissolved in it five decigrams as long as I used 5 per cent. solutions; now I dissolve in ten grams of the same sublimate solution three decigrams of cocaine hydrochlorate, by which I obtain a three per cent. solution of cocaine hydrochlorate. A drop of this contains three milligrams of cocaine hydrochlorate.

To facilitate the preparation of this exact sterilized cocaine solution he uses vials with a wide neck, which will contain ten grams of fluid exactly. The vial is filled with the sublimate solution already mentioned, 1 to 5,000. Then add three decigrams of cocaine hydrochlorate, which is weighed in paper cases. By shaking the solution it is made ready for use in a few seconds. The piston rod of the syringe is divided into ten parts, so that one stroke discharges one decigram of fluid, or a drop. The injection needles have different lengths, ten and twenty millimeters respectively, in order to be able to adapt them more easily to the different dimensions of the upper and lower jaws. The method of inserting, or direction of the needle, he considers of the greatest importance. He says: "If I direct the point too much against the epithelial side the uppermost layer of the epithelium is lifted up by the slightest pressure on the piston rod, in the form of a transparent blister. Such injections are of no value at all. But if I put the needle too much toward the jaw, then I discover that I have struck the socket. I may press as long as I like and not get anything out of the syringe. The piston rod remains immovable, and in trying to overcome the obstacle by forced exertions either the piston rod or the glass cylinder breaks, as I have sometimes experienced. I must therefore, hold the syringe after the manner of a writing-pen, and force it between the periosteum and the socket. At this place the injection fluid is pressed by the tension of the periosteum into the bone tissues of the alveolar wall, and from there into the periosteum. The injection proves to be successful by the gums growing more and more pale, and the formation of a transparent circumscribed blister which resembles, very much, a ranula swelling.

"Four horizontal and four vertical punctures of one drop each generally suffice. At the mesial labial papilla of the gums I also inject, two or three millimeters distant from the gums, pricking toward the distal labial edge. If the injection has succeeded well after the first horizontal puncture, of which the growing paleness of the gums is a sure criterion, the next puncture being made in the pale place, it is not felt by the patient."

He said many authors advise delay from five to ten minutes after the injection has been made before extracting the tooth. I consider this superfluous for the anæsthesia, and I extract immediately after the injection, and to this proceeding I ascribe the circumstance that I have had to experience comparatively very few and very slight accidents.

Intoxications after cocaine injections only occur in my experience if of 20 or 10 per cent. solutions half, or even a whole syringeful has been injected. In this case, if the solution be 20 per cent, either one decigram or two decigrams of cocaine hydrochlorate has been injected; but with 10 per cent. solutions, five centigrams, or one decigram of cocaine. These are doses which are too high under all circumstances. I only had instances of real intoxications in the beginning of my cocaine injections, and as long as I injected such doses. Now, when using only 3 per cent. cocaine solutions I have but rarely perceived reflex symptoms, never real intoxications.

With persons of nervous disposition the dentist must try to quiet their excitement by showing himself perfectly composed and at ease, in order to gain their entire confidence and trust. Having thus succeeded the whole procedure may be properly executed. With a 3 per cent. solution one can only experience nervous reflexes, which, however alarming they may seem, have nothing dangerous in themselves.

In the discussion which followed the reading of these papers Dr. Roberts said that he had had analyzed samples of many of the patented nostrums now on the market for "painless dentistry" and found that they all contained from 1 to 3 and even 5 per cent. cocaine, antisepticised by means of carbolic acid; so large a proportion of the latter in many of them that sloughing of the gums, or necrosis of the process, would follow their use. The most recent of these preparations contain a small per cent. of tropa-cocaine. He considers that the main thing now is as to the effect of the different antiseptics in connection with cocaine, because it seems to be absolutely settled that a 2 to 4 per cent. solution of cocaine will do the work harmlessly.

Dr. Pruyn gave the results of his long series of experiments with cocaine, locally and hypodermically, of hundreds of cases in actual practice, and also his experiments upon the lower animals. As the result he concludes that for hypodermic injection a 2 per cent. solution with about a half per cent. solution of salicylic acid to preserve it, answers just as well as a 4, or 6, or 8 per cent. solution. He said, I have used, in some cases, hot water simply, thinking that possibly there might be something in the pressure of the liquid of the solution of cocaine upon the ends of the nerves that had something to do with obtunding, and it has done wonderfully well. I dare not say it is just as good as cocaine, but in the few cases I have used it, it has proven that it is worth something. I would like those who are experimenting in this line to try it, and find if we can get any results from it.

Dr. Cheney: I have tried tepid water numerous times and find it very effective. If the patient does not know the difference it is just as good as cocaine.

Dr. Parker: Isn't that a hypnotic effect? You might just as well use cold water. I would like to hear from some gentleman who has experimented with cocaine on abscessed teeth, or one that was abscessing.

Dr. Freeman, Chicago: I have used it in abscessed conditions and my results were very unsatisfactory unless I went beyond the line of inflammation; then it proved satisfactory.

Dr. Parker: The only way I have had good results in abscessed teeth with cocaine is to first spray the tooth slightly, then inject the cocaine thoroughly into it, as the paper states. If you can start at a point, force the cocaine along and keep following it up, inserting the needle at the point which is anæsthetised, you can relieve the pain, but if you insert the needle into highly inflamed tissues you will cause the patient more pain by the first injection than you will by the extraction of the tooth.

Dr. Cravens expressed regret that, so far as heard in this discussion and the papers read, the use of cocaine in dentistry would seem to apply only to extracting teeth. He said: I hope Mr. President and gentlemen that we are not degenerating into a professin of tooth-pullers. Local anæsthesia is beneficial for other purposes in dentistry than simply the extraction of teeth. I have had some experience in local anæsthesia by the use of cocaine in the operation of scraping the roots, or operating in the pus pockets of pyorrhœa alveolaris. I apprehend the operation without anæsthesia is about as painful and certainly much more protracted than that of extracting a tooth.

My method is to make a saturated solution of the hydrochlorate of cocaine in chloroform, making something more than a 6 per cent solution after you get the solution saturated. Hot water is necessary in my method. By the application of cocaine without preceding it with water I do not get the results. I do not succeed in reducing the sensibility of the surface of the root.

I precede the cocaine application in the pockets by washing the pocket with the hot water, and I use water just as hot as I can get it there and not burn, just short of the point of scalding. I use the ordinary little syringe that holds, I suppose, half an ounce, and I discharge one, two, three, perhaps four charges from that syringe into the pocket before I apply the cocaine at all, then inject a single drop of cocaine.

The first application of cocaine is guarded; that is, I keep the saliva away and protect it for perhaps two or three minutes, then I wash again and apply the cocaine. I make three applications of cocaine in succession, sometimes four. After the third application, in most cases, and certainly always after the fourth, I can go to the end of the root—the apex, if the pocket goes that far—and can scrape the root, as I have done in some cases, until my conscience revolted, because I thought I was hurting the patient, and I have been told there was no pain felt.

It requires about fifteen minutes to make the three applications; and four will do it in every case that I have encountered.

Now, one other point, in regard to cocaine escaping about the mouth. If, in making these injections, the dentist will place some absorbent cotton around the tooth and pack it well about the sides, it will prevent overflowing. In case it does escape and the patient experiences the symptoms of loss of the sense of taste, stiffness of the cheeks, etc., I dissipate that in from one to two minutes by simply rinsing the mouth with alcohol or whiskey.

Dr. Hewitt: I can corroborate what the last speaker has said with regard to the application of cocaine in pyorrhœa alveolaris. Cocaine should be purchased in large crystals because if it is purchased in fine powder you will almost always find it adulterated, scarcely ever pure. The prisms should be in their primitive rhomboidal form, clean and large; then they should be pulverized very thoroughly. Dip the point of a moistened instrument in this pulverized cocaine and carry it to whatever point you choose and the effect is almost instantaneous.

I am in the habit of using it in that way in the treatment of pyorrhœa alveolaris. I carry cocaine upon the point of instruments up into the pockets, and by moving the instrument around on the gums, with very excellent effect.

There is a large range of application where cocaine is exceedingly useful, but I feel it my duty to the profession to call attention also to its dangers as a hypodermic injection, unless the injections can be localized, or the effect counteracted by a heart stimulant.

Dr. V. Paredas, Bogota, Colombia, finds that a local application of cocaine, made by wrapping or binding around the tooth a bandage bathed in a 10 per cent. solution of cocaine with antipyrine and allowed to remain ten or fifteen minutes will produce a good result.

Dr. Aguilar, San Jose, spoke of the use of cocaine in preventing nausea while taking impressions; rubbing over the palate a solution, and so anæsthetizing it partially, taking an impression without nausea or producing vomiting, which without it in many cases is produced.

To obviate the unpleasant taste of the cocaine, he says, his custom is to mix with the solution a very small quantity of saccharine in the proportion of fifty centigrams of water to three of pure cocaine. Take a very small amount, just what you could get upon the point of a penknife, of saccharine, and mix it with the cocaine, and thus obliterate the bitter taste of cocaine almost altogether.

Dr. Fletcher, St. Louis, advises that the tissues be tested with a sharp instrument, and the extraction made as soon as possible, before the cocaine is taken up into the circulation. The bleeding that ensues eliminates the cocaine with the effusion of the blood, so that you get but very little into the circulation, and it cannot possibly get as far as the heart, at least not enough to do any particular harm.

For the painless removal of pulp tissue Dr. Rose rubs small portions of the cocaine in a mortar or on a glass with a single drop of carbolic acid, touching the exposed nerve with what can be carried of this solution on a nerve broach.

Dr. Hewitt accomplishes the same result with a paste of cocaine and glycerine. He said, I would use the glycerine in preference to carbolic acid; you can take that paste, apply it to the bare nerve if you can get to it, then follow it clear down to the apex, and you can remove it without a particle of pain.

Dr. Wm. N. Morrison protested against the extraction of so many teeth, and said, I hear with regret the essayist call attention to the fact that he extracted two central incisors, and two lateral incisors, and two cuspids, by his hypodermic use of cocaine, for the insertion of an artificial denture, in this age of modern crown and bridge-work. We should counteract the detrimental influence that has been produced, and is being produced by the advantage claimed to have been gained by the painless extraction of teeth, for I believe, from an anatomical or physiological standpoint, in the crime next to abortion comes the ruthless extraction of human teeth.

Dr. Pruyn spoke of the cases in which it would be unwise to use cocaine, for instance, in a case of known pregnancy, or where there is any known pulmonary trouble, or where there is a diseased condition of the kidneys, because cocaine has a very marked effect on the kidneys. He said, I find by experience, the best results attend its use after the patient has had a full meal; I have found the toxic effects much more marked when used upon an empty stomach. If I wanted to use it in a case of any considerable extent I should first fortify my patient by the use of morphia. To those of you who want to use it I should advise first to experiment upon dogs and you will learn the symptoms that you cannot see unless you bring your patient down to death's door.

Dr. W. C. Davis, Lincoln, Neb., read a paper entitled, "Obtunding the Sensibility of the Dentine."

He said, if we are to eliminate pain from an operation we should first study its cause, and the means by which it is transmitted to the seat of realization. He combats the theory of nerve fibers in the dentine, claiming that the tubuli are filled with protoplasm, which is capable of transmitting the sensation of irritation to the odontoblasts lining the pulp chambers and root canals, each of which is connected with one or more of the terminal fibers of the sensory nerves. Chloride of zinc, or carbolic acid applied to the dentine will coagulate the protoplasm, thus destroying its life and obtunding sensibility, because we have destroyed the medium through which the sensation is transmitted. Coagulation of the protoplasm not being permissible because of jeopardy to the pulp, the alternative remains of removing the protoplasm from the tubuli. This he accomplishes through the application of alcohol vaporized by hot air, leaving the tubuli empty and the sensibility of the dentine obtunded. But as the dentine is hygroscopic—capable again of taking up moisture; from within by capillary attraction, from without by contact with the atmosphere; the next step is to fill the tubuli with some other substance. I fill the tubuli with a resinous gum in suspension in a volatile oil. Almost any one of the essential oils will answer the purpose. Upon the application of hot air the oils are easily volatized, and if it has been diluted slightly with alcohol it will readily go into the tubuli as far as they are empty, leaving the gum to fill the space. To this distance you can excavate with impunity; consequently, the success of the operation will depend upon the thoroughness with which you extract the protoplasm.

Dr. Davis then reviewed other methods resorted to and said, I believe there is no method which has partly obtunded the sensibility of dentine, whether freezing, superheating, application of caustics or devitalizing agents, etc., that cannot be explained upon the hypothesis given. This method of obtunding the sensibility of the dentine will come nearer attaining success than any before presented, inasmuch as a foreign substance is inserted in its place after extracting the medium for transmission of pain.

In cases where local means seem to meet with failure a general anæsthetic is resorted to, as follows: Take an ordinary gas apparatus with a Buffalo inhaler. In the inhaler place one drachm each of alcohol and ether, also four or five drops of nitrite of amyl in solution. Administer as in giving nitrous oxide. The first effects noticed are those of the gas. Five or six inhalations of this compound anæsthetic are sufficient to produce anæsthesia. The symptoms are similar to those after the administration of nitrous oxide gas alone, with addition of the flushed face caused by nitrite of amyl. The anæsthesia from the gas and ether soon passes away, leaving the patient semi-conscious, with the power of conversing with the operator and seemingly capable of realizing all that is going on; but there seems to be a temporary paralysis of the fifth nerve, especially the third division, lasting from ten to fifteen minutes, sometimes twenty. It is a fact well known to the medical profession that a few inhalations of nitrite of amyl will give speedy and temporary relief to neuralgia; also the suffocating sensation incident to an attack of asthma; also that upon its administration there seems to be dilatation of the capillaries in the parts supplied by the fifth nerve, as is evinced by the flushed face; altogether going to show that this drug seems to have a special action on the fifth nerve. This led me to experiment, with the result above stated. These two methods will make the operation of tooth-extraction entirely painless, the result being universal as far as my experiments have gone at present.

For general practice I would not, at present, care to advise the use of any general anæsthetic for an operation upon the teeth. It is certainly attended with some danger. The amount of danger connected with the administration of an anæsthetic as complicated as the one just given I am not now prepared to state. However, I believe it to be comparatively safe as the action of the various ingredients upon the cardiac muscles and regions, as well as upon the respiratory organs, is antagonistic.

This paper was passed without discussion and the section adjourned to 2:30 p. m. Wednesday.

SECTION 5. DENTAL AND ORAL SURGERY.

In the absence of the chairman of this section T. W. Brophy, the Secretary, Dr. L. P. Dotterer, of Charleston, S. C. opened the session.

M. H. Cryer, of Philadelphia, read his paper on "The Surgical Engine and Its Uses."

Dr. Cryer first outlined the requirements of *the ideal* surgical engine, pointed out the imperfections of those now in use, and said: Last winter I consulted Mr. C. Doriot, who suggested a new engine which should have greater power and better mechanical arrangements than those now in use. The S. S. White Dental Manufacturing Co. gave him facilities to work out ideas about which we had mutually agreed, and I have great pleasure in introducing the result to the profession in this meeting. The general appearance and construction of the engine proper is similar to the Bonwill, though it is a much stronger machine, while its mechanism is of a higher order. It has been completed only a few weeks; too late for an opportunity to use it in the hospital clinic, but its work as tested upon dead bone is fully satisfactory.

Dr. Cryer then described some typical operations in which the surgical engine is most essential, as for the removal of sequestra, caries, osteomata, for cutting into and through bone for the resection of nerves.

He said: The proper amputation of the nerve at the ovale foramen is one which involves no little difficulty, at times, by reason of the hemorrhage and the limitations imposed by the surrounding tissue, which interfere with direct vision. The bony structures offer an unyielding resistance to the use of any of the special instruments which have hitherto been used for the purpose. To obviate the difficulties which I have noted, and to furnish a means for the amputation of the nerve with certainty and precision at the exact point desired, [in the case under consideration the foramen ovale] I have devised an instrument which I call a neurotome. It is constructed of steel and consists of an outer tube with a fenestrated end, the free extremity of which is rounded or tapered, and having on its inside surface near the end a projecting shoulder, which is continuous around the inner periphery of the outer or canular tube. The inner cutting portion of the instrument consists of a steel spindle, which fits closely and smoothly within the outer tube, and has its free end fashioned into a tubular knife similar to a cork-borer or leather punch. In use, after the nerve has been dissected out from its canal, its end is passed through the distal opening of the neurotome and out through the fenestrum in its side. The instrument is gradually pushed along the nerve and worked in the direction of the foramen or exit. When it has been ascertained that the end of the neurotome has been brought into close contact with the bone at the foramen and the nerve is made tense by traction, the central cutting-punch of the neurotome is driven home with a rotary motion against the shoulder, mentioned before at the end of the outer canular tube, which will cleanly sever the nerve at the point desired, with no risk of injuring the deeper structures, or the adjacent blood vessels.

Dr. Cryer gave the history of a case in which eight distinct and separate operations by some of the most skillful surgeons had been performed for the relief of neuralgia in the region of the lower jaw without relief, the pain becoming more and more aggravated.

In the finally successful operation the nerve was drawn out of the canal and cut off as high and near to the ovale foramen as possible. Upon the section of the nerve which was removed there were two large and distinct neuromata, the lower one being impinged upon by the triangular process of bone at the opening of the inferior dental canal; the upper one was under the external pterygoid muscle. Beyond a doubt here was the cause of the neuralgia. Several arteries were ligated before the parts were closed. The patient came from under the influence of ether without the slightest pain and has not had a twinge of any kind whatever up to this time. It is now over nine months since the operation was performed.

Other operations named were: Resection of the elbow joint, resection of a portion of the ramus near the angle of the inferior maxilla, in order to make a false joint to relieve a true ankylosis of the temporo-maxillary articulation.

The removal of a diseased portion of the calvarium measuring three by four inches without inflicting any injury to the membrane of the brain, osteoma from the floor of the orbit, osteoma from the inferior surface of the body of the sphenoid bone. This tumor filled up the posterior of the nares and the greater portion of the naso-pharyngeal space.

Caries of the mastoid process of the temporal bone. For the relief of coxalgia by removal of the coccyx, through an external opening, without disturbing the perineum.

Treatment of alveolar hypertrophy; for freshening the edges of a recto-vaginal fistula. In freshening the edges or surface of soft tissue, coarse corundum-wheels or stones are used, with a free flow of water running over the parts. I have recently received two communications relating to the use of the surgical engine in the removal of large calculi from the bladder, the first from Dr. Bonwill and the second from J. G. Kerr, M.D., LL.D., of Canton, China, who is a specialist in this line of surgery.

It must be seen that the surgical engine has a wide field of influence before it, not alone in bone surgery but in that of other tissue as well.

As yet it has not been much employed for operations in brain surgery, but it is to be assumed that the time is not far distant when it will be universally used whenever the brain-case is to be opened.

At the conclusion of this paper Dr. Cryer explained the operation of his new engine and gave practical illustrations of its work. He showed the applicability of the engine for many uses in general surgery, and said it was especially useful in operations for trephining the skull. One of the beauties of the engine is that in removing the inferior maxillary bone, and cancerous growths, it is possible to remove the diseased bone down to the periosteum, and the bur which removes the bone will not cut the soft tissues. In this way all the diseased parts can be removed much better than by the old method of using the chisel and the mallet, which would break the bone and injure the periosteum and soft tissues. In working in the back part of the mouth there is no danger whatever of cutting the soft tissue, while it will perfectly cut all of the bone. By using corundum-wheels under a free flow of water, soft tissues can be cut away.

The paper was discussed by Drs. Jno. S. Marshall, W. C. Barrett, Thos. Fillebrown, M. H. Cryer, and T. W. Brophy, who arrived at the conclusion of the reading.

Dr. Cryer argued in favor of cutting off the nerve; Drs. Marshall and Barrett favoring evulsion. Dr. Fillebrown thought in tearing out the nerve the contraction of cicatricial tissue would bring the fibers together, producing pain by pressure over a large space, which would not be the case after a smooth cut; the latter method being therefore physiological, scientific and right.

Dr. Brophy spoke of the re-formation of nerve tissue, and thought the engine bur would plow up the surface of the nerve-canal in the bone, which would cause an exudation and permanently plug up the canal, making it impossible for the nerve to re-form.

At the conclusion of this discussion the section adjourned to 3 p. m. Wednesday.

### SECTION 6. OPERATIVE DENTISTRY.

After an address of welcome from the Chairman, Dr. Wm. Jarvie, M.D.S., of Brooklyn, N. Y., Dr. Henry W. Morgan, of Nashville, Tenn., Secretary of the Section, read a paper by Dr. H. L. Ambler, of Cleveland, Ohio, entitled "Tin Foil for Filling Teeth."

The advantages claimed for tin foil by Dr. Ambler are that it wears longer than oxychloride or gutta-percha; that it is quickly and easily manipulated; that being soft and ductile it closes the dentinal tubuli, being driven into them; that from its low conducting power it can be used near the pulp, and that dentine often hardens under it. He claims that it is especially useful in chalky teeth, and that it lasts long in buccal and palatine cavities.

Strips of No. 10, from one to three thicknesses, can be welded together, cohering as well as semi-cohesive gold, or better, and can be manipulated much more rapidly; therefore, if desirable you can produce any contour.

Instruments with square ends and sides, and medium serrations, are best adapted for hand force, and the majority of medium serrated hand mallet instruments will work well on No. 10 tin of one, two or three layers, using a four-ounce mallet with a fair, steady blow. Generally cavities are prepared the same as for gold, except that the grooves or pits should be a trifle larger. Many cavities can be filled with less excavating than is required for gold, and some approximal cavities in bicuspids and molars can be filled without removing the masticating surface. Here, especially, the cavities should be cut square into the teeth so as not to leave a feather edge of tin when the filling is finished. In nearly all approximal cavities in bicuspids and molars you will find some form of matrix of great advantage. By driving the tin firmly against the matrix you secure a well-condensed surface, and the teeth will move apart slightly, so that with a bevel or thin plugger you can force the tin between the matrix and the edge of the cavity and thus be sure of having a tight filling and plenty of material to finish well; then after removing the matrix condense with thin burnishers and complete the finish as for gold.

We find that tin prevents further decay at the cervical margin of deep cavities oftener than any other metal or combination of metals. We fill from one-fourth to one-half of the cavity with tin, completing with gold when the tooth is of good structure, which gives all the advantages of gold for a masticating surface.

In the discussion of the paper Dr. E. T. Darby said: The author of this paper has certainly paid a very high, and I don't know but a very worthy tribute to tin. I have always said that tin was one of the very best filling materials we have. I believe more teeth could be saved with tin than with gold. Whether tin possesses the antiseptic properties in as great a degree as is claimed by many I sometimes question, but I do know that it has a saving quality that we do not always find in gold. There is but one disadvantage that tin possesses so far as

I am aware, that is its color, but in all approximal cavities that are exposed to view, I believe the average dentist will do as well with tin as with gold. I believe if the dental profession would use more tin they would save more teeth. For children's teeth I know of nothing better for masticating surfaces.

Madam Tiburtius Hirschfeld, of Berlin, heartily indorsed the use of tin and gold after a practice with this material for twenty-four years. Her practice has been mainly for children and ladies, and she thinks for filling children's teeth there is no better material than tin and gold; sometimes these fillings were put in when the child was seven or eight years old and at the age of seventeen the fillings were still perfect.

Dr. R. R. Freeman, Nashville, Tenn. You don't know how happy I feel to hear the subject of tin foil brought up before this Congress. I learned something of tin in the early school, when I had the honor to be upon the stage with Madam Hirschfeld, when we received our diplomas. I have written upon tin foil, I have talked upon it and advocated its use. I know what it is doing, and I know what it has done for twenty-five years. All through our southern country we have those who are using tin foil for its therapeutic properties; it has that healing property, for the dentine of children's teeth, that hardens them, and it has been within a few years that one of our best practitioners said, "I must acknowledge that tin does save teeth." I am glad to give my testimony in behalf of tin foil.

Dr. Albert H. Brockway, Brooklyn, N. Y., said: If you are to do the best thing for the patient be extremely eclectic in practice. It is for us to determine what to use for a given case under given conditions. I am a strong believer in the use of tin foil in such cases as will admit of it; I use it more or less, and I use it for two reasons especially. The first is from its adaptability, and facility with which a saving filling can be made in favorable cases; I am also inclined strongly to believe in its therapeutic properties.

I use it especially in children's teeth, in cases where tin foil has been strongly recommended, and in which recommendation I quite agree; but there are many cases in children's teeth where it seems to me that tin foil could not be used so successfully as other materials, notably gutta-percha.

Dr. Gordon White, Nashville, Tenn: I claim for tin, after having used it for nine years, that it is the best filling material that has yet been given our profession, excepting that it will not stand friction; I think it is the best tooth-preserver we have.

Dr. C. S. Stockton, Newark, N. J., spoke of the many patients who are unable to pay the large fees incurred by gold fillings, and said, it is necessary and right to save the teeth of those who are not able to pay the large expense of gold-work, and if we have a material that will save teeth it seems to me it is our duty to use it. Tin is one of the best materials for saving teeth, and we should use it more than we do.

Dr. St. George Elliott, London, England. It is to me a very great pleasure to return to this country and find that tin is, at least, beginning to have a large number of advocates. In Europe we all look upon Dr. Abbott, of Berlin, as the father of modern dentistry there; he was one of the earliest, though not the first, to use tin foil, and he did so very successfully; he carried it out in his own practice, and his son-in-law, Dr. Miller, took it up, as did also, Dr. Jenkins, of Dresden. For ten years I have used it very largely; I have averaged from four to five or six fillings a day. The greatest advantage of tin and gold has not been spoken of; you know if you get a preparation of tin and gold in correct proportions there is practically a chemical union between the two, and you get not only hardness but a certain amount of expansion. It is exceedingly valuable in filling crown cavities of molars. Its hardness is not immediately gotten; it takes from one to three years to harden.

Prof. James Truman, Philadelphia, Pa.: It is with a great deal of gratification that I find even at this late day, after forty years of practice in the use of tin foil, that it is coming up again with honor. I have long been satisfied that the profession has lost much in the abandonment of this material. In regard to tin and gold I have used it a good deal, and have seen Dr. Abbott operate with it in Berlin, and I know a good deal about Dr. Miller's use of it, and I am satisfied that it is a most valuable combination. It can be placed in wet, and if it is placed in wet it is better than when dry, owing to the action of the fluids of the mouth producing galvanic action between the two metals, that produces hardness. I remember once I had occasion to remove an anterior approximal filling of Dr. Abbott's, and I found that the tin and gold was as hard as any amalgam filling I ever saw, and I had great difficulty in cutting it out; that was due to galvanic action between the two metals.

Dr. W. W. Freeman, Chicago, spoke of the value of tin fillings finished with cohesive gold, and said, I indorse the use of tin and gold, and especially do I believe in its chemical action.

At the conclusion of this discussion the Section adjourned to 2:30 p. m., Wednesday.

SECTION 7. PROSTHESIS AND ORTHODONTIA.

This Section was called to order at 2:30 p. m., by the chairman, Dr. C. L. Goddard, San Francisco, Cal., who read an address of welcome.

Dr. V. H. Jackson, New York, then read a paper entitled, "A Method of Constructing Spring Appliances for Correcting Irregularities of the Teeth."

This paper was very fully illustrated, Dr. Jackson having about forty drawings of appliances, he also announced that models of many of the cases would be shown to the Section at another time.

Dr. Jackson defined a "regulating appliance" as being, "any apparatus designed to move natural teeth that are out of the line of harmony into their proper position."

In constructing an appliance for this purpose the first requisite is, that it shall be sufficiently well anchored to withstand either the constant or interrupted force necessary to correct the irregularity without being materially changed in its position. The comparative value of the different systems that have been presented for this purpose can only be satisfactorily understood by having had experience with each of them.

In the system introduced by Dr. Jackson the appliance is constructed of wire, with a base wire, which is so termed on account of its being the foundation portion of the regulating appliance, to which "cribs" that clasp the teeth for anchorage are attached, and also springs that are to cause pressure to correct the position of those teeth that are out of line.

The base wire can be made of any metal desired. Metal used for this purpose alone should not be springy, but stiff and unyielding. It can be made any shape, round, square or flat. The round is usually preferred.

A perfect plaster model of the teeth should first be made and carved, especially the gum portion, at the necks of the teeth to be used as anchors.

The cribs, forming a partial clasp, are made, preferably, of gold or german silver, or any other metal applicable, about No. 33 to 36 standard wire gauge (Brown & Sharp) in thickness, hollowed and shaped to fit accurately the contour of the teeth that are to be used as anchors, and arranged to press well up about the neck, and at the same time made to curve sufficiently over the prominences of the tooth toward the grinding-surface to prevent the appliance from pressing on the gum. The partial clasp is arranged on the side of the tooth to which the base wire is to be attached.

If adjoining teeth are to be used as anchors, partial clasps should be arranged on each so as to touch each other at the junction of the teeth.

A spring wire, about No. 22 standard wire gauge, or a little larger, as the case may require, is formed so that it will fit the labial side of the tooth to be moved, with both ends passing over the arch at the junction with the adjoining teeth, and curved about the lingual side near the gum line, to rest on the metal described, but it should be made to fit loosely, so as not to injure the plaster model in removing it.

The clasping power of the crib depends much on the spring properties of the metal used for the springs. Piano-wire is at present the most efficient, although spring gold, German silver, and iridio-platinum wires are often utilized. When the appliance is adjusted in the mouth, proper pressure is applied by making the necessary changes with the clasp-bender.

The uniting of the metals of the crib to the base wire is accomplished by soldering either with soft solder or silver solder.

Soft solder is used for attaching the parts constructed with piano-wire. If silver solder is to be used, the parts should be held in position on the model and united with hard wax, after which they should be removed together, and invested sufficiently with plaster and sand to hold them in position while soldering. It is desirable usually, after the appliance is inserted in the mouth, that it should remain untouched for about three days, so that the patient may become accustomed to it. After that time, the pressure may be changed once a week, or oftener if desired.

Pressure is increased by removing the appliance and bending the spring in the direction in which the force is desired.

A detailed description of the method as applied to the correction of typical and more commonly occurring forms of irregularities was given, in connection with an elaborate series of illustrative charts. These will appear in full in the Congress Transactions.

In the discussion which followed, Dr. Jackson, in reply to a question, said that if patients kept their teeth clean they could prevent the wires from corroding. If it were found that a patient allowed it to rust it would be necessary to see the patient more frequently, and see that it is kept bright. This can be done by scraping spots of rust off till the surface is quite bright, when it will generally last as long as the necessity for it exists. It should be kept bright, even if necessary to polish it every day.

He had found nothing so good as piano-wire, having tried gold wire and iridium and gold. These are good, but as soon as you apply heat to hard solder you destroy all the spring and spoil the virtue of the wire.

Dr. J. E. Keener asked—:

1. Do you ever fail to move a cuspid? We all know that in the case of one well advanced in years it is very difficult to do this.

2. Do you rotate and at the same time and with the same appliance move the tooth backward or forward?

Dr. Jackson said that in regard to moving an incisor and at the same time rotating it, it requires only sufficient thought and study to devise and shape a spring so that by shortening or lengthening a loop, motion can be diverted in any desired direction.

Each case must be studied as to the peculiarities presented, and the appliance made to suit.

Dr. Allwine thought that in some cases caps would be preferable to bands.

Dr. Jackson replied that he did not use caps, as they sometimes caused the opening of the bite, and he considered it very necessary to keep the teeth in exactly the natural position.

Dr. E. M. S. Fernandez, Chicago, thought the paper was invaluable, but he differed with it on two or three practical points. He disapproved of letting any patient know how to take the regulating appliance out, because, especially in the case of children, if they can remove it they will do so, and only return it when they come to see the dentist; in this way the appliance will not do any good, and the dentist will get the blame for the failure.

As to cleaning the rust from steel wire, his way is to throw it into alcohol; he learned this from seeing a piano-tuner clean the rust from the wires of a piano, and now he always throws every such appliance into alcohol whenever he removes it from the mouth; then brush it off with soap and water, and the appliance will not rust readily again.

After some further discussion by Drs. Prosser, J. Rollo Knapp, E. Marshall Smith, M. R. Finley, C. S. Case, C. L. Boyd and others; Dr. C. G. Myers, of Galveston, Texas, read the following paper: "A method of Fusing Porcelain Facing to Backing and Cap."

The great objections to porcelain-faced crowns have been, in the past, the liability of the facing to fracture, and lack of cleanliness. To overcome these defects I have succeeded in fusing in the porcelain facing to the backing and also to the cap at the time of soldering.

The material employed is the white enamel used by jewelers in ornamenting gold. This comes in lumps almost as hard as flint, and is reduced to an impalpable powder by grinding in water in an agate mortar, and afterward washing thoroughly. After fitting the thin pure gold backing as accurately as possible to the facing remove backing, and with a fine-pointed brush place a small quantity of the enamel; mix with water to the consistency of cream on back of the facing, but do not let it come in contact with the pins. Replace the backing, and bend pins slightly to hold it in place; then place facing on asbestos, and flow up backing with solder. The heat required to flow the solder will also flow the enamel, forming a perfect union between porcelain and gold. In like manner the enamel may be used to fill the space between facing and cap, and when soldered it will form a perfectly cleanly as well as strong piece of work.

The enamel can also be used in gold plate-work when the teeth are to be soldered to the plate, thereby doing away with the uncleanly joints so often found. By removing the objectionable features of work we thereby widen its scope.

In this short paper I have tried to give you the results of some experiments I have been carrying on for several months. If they can be of any service to you our time has not been vainly spent.

Adjourned at 5 p. m.

### SECTION 8. EDUCATION, LEGISLATION AND LITERATURE.

The meeting was called to order at 2:30 p. m. by the Chairman, Dr. J. J. R. Patrick, Belleville, Ill.

The Secretary, W. H. Whitslar, M.D., D.D.S., Cleveland, Ohio, read a paper by P. Macarovici, M D., Jassy, Roumania, on "The Status of the Art of Dentistry and of Dentists in Roumania."

Dr. Macarovici said that after searching in vain through the literature of Roumania for anything whatever bearing on the subject of dentistry he had endeavored to form a dental society; the object of which should be to diffuse a knowledge of chirurgico-dental science in the Roumanian language; to establish a special school of dentistry; to found a dental museum, and to establish a dental library. The total failure of this great undertaking was a source of bitter disappointment to him. Of the status of dentistry in Roumania he says: "An evidence of the slight degree of confidence which the Roumanian public reposes in dentistry may be gathered from the circumstance that the native dentists might exhibit mountains of human teeth if the patients were not in the habit of demanding the same after extraction for preservation among their relics. Our patients will have nothing but extraction; of re-implantation, filling, or similar chirurgico-dental operations there can be no question in our country, for even when we have succeeded, after prolonged discussion, in persuading a patient to submit to filling he demands the impossible, namely, that the entire operation shall be concluded instanter.

Up to the year 1888 the only requirement from those not holding a foreign diploma was that they should be able to pass the "Subsurgical" examination. At this examination he was compelled to show that he had become practically acquainted, by a year's attendance in a hospital, with the following procedures and appliances: venesection, leeching, blistering, clysters, cataplasms [fontanellen offnen.], vaccination and the extraction of teeth. The candidate, besides, was required to be able to read and write.

In 1888 a dental law was enacted by which the standard was materially elevated, giving grounds, in the words of Dr. Macarovici, "To hope that the country may shortly be able to show more worthy representatives of the dental art than it now possesses."

This paper, owing to its purely historical nature, called forth no discussion. On motion of Dr. Noble this paper was passed and that of Dr. Frank W. Sage, of Cincinnati, Ohio, entitled "The Editorial Function in Dental Journalism," was taken up and read by the secretary of the section owing to the absence of the writer.

Dr. Sage, in this paper, indulges in severe criticism of the editors of dental journals who "load their papers with swelling phrases which mean nothing in particular, or are not infrequently mere paraphrases of familiar passages to be found in standard dental works," who "publish papers without question as to their character or worth," who "employ a stenographer to report the discussions, and insist on having a full, copious report made, printing the exact words published without revision or emendation, promulgating on the printed page the wildest theories, the baldest speculations of Smith, Brown, et al., since those theories and speculations had furnished a theme for discussion in the convention.

"Eminent men in the profession, editors and others, years ago called attention to the fact that our literature is not of the high order which should characterize a profession so progressive in other respects as ours. No one, to our knowledge, however, has suggested the expediency of the editors themselves taking a higher stand so as to be in a position to stimulate contributors to worthier efforts."

Dr. Sage also criticises the lack of *editorial matter* in the dental journals and also the lack of *editorial revision* of contributed matter. He said, the editors of the great secular magazines do no hesitate to suggest, even to writers of distinction, changes in manuscripts submitted to them. True, they stand in an exceptionally independent attitude. Few authors feel that they can afford to ignore the editor's suggestions. Editors are supposed to know best what the public want. Authors usually defer to their opinions. Now, why should not the same deference be made to the opinions of dental editors? Why should they not be sought out and consulted in this matter of deciding what amendments are required in any manuscript which it is proposed to print and bring to the profession's notice? Has the time arrived for dental editors to take a higher stand in this matter? Will it not be demanded of them by their readers in the near future? What would be the probable effect of putting into operation this suggestion? First, the contributor would be stimulated to self-improvement as a writer. Secondly, the editor would be put upon his mettle, and possibly stimulated to more worthy efforts in his own writing. It would further serve to create a distinction between such journals as aspire to lead and instruct their readers, and others which are obviously mere mediums for advertising dental wares. It would undoubtedly increase the journal's circulation, for unquestionably the improvement would not be overlooked by the most casual reader. The effect at first might be to check production. That might not be an unmixed evil. Let us cut out all irrelevant matter from our journals; let us put a premium on brains, and then trust the future for results. Place a few salutary restrictions upon contributors. Pay something for contributions. Payment for an article is a guarantee of real worth in the article accepted.

Of book reviews, he said: What more important service can the editor render the large and increasing number of purchasers of dental works than to give them, in advance, an impartial judgment of their merits and defects alike.

In the opinion of Dr. Sage it is in the published reports of discussions in our conventions that the need of editorial revision is most palpable. Many of these reports could be cut down one-third or one-half with very desirable effect. Of course, much latitude must be allowed our extemporaneous speakers in conventions. They must be left untrammeled to range over the field of the subject as the inspiration of the moment dictates, but after an experience of twenty years in short-hand reporting in dental conventions, the writer of this paper is of the opinion that but few speakers appear to any advantage in a verbatim report. They appear better in print in a synoptical report.

In conclusion he said: Let us hold up the hands of the editor and make him the central figure; let us concede to him the right and privilege of infusing more of his individual spirit into the journal, even if it be at the expense of the contributor's ideal of what the reader wants. Thus shall we accomplish the desired object of conferring upon the journal a distinctive character, calculated to extend the range of its influence and fix the standard of its authority.

There was no discussion on these papers and the section adjourned to meet at 2:30 p. m. Wednesday.

[*To be continued.*]

